# Discoloration and Surface Changes in Spruce Wood after Accelerated Aging

**DOI:** 10.3390/polym16091191

**Published:** 2024-04-24

**Authors:** Jozef Kúdela, Pavel Ihracký, František Kačík

**Affiliations:** 1 Department of Wood Science, Faculty of Wood Sciences and Technology, Technical University in Zvolen, T. G. Masaryka 24, 960 01 Zvolen, Slovakia; kudela@tuzvo.sk (J.K.); p.ihracky@kronospan.sk (P.I.); 2 Department of Chemistry and Chemical Technologies, Faculty of Wood Sciences and Technology, Technical University in Zvolen, T. G. Masaryka 24, 960 01 Zvolen, Slovakia

**Keywords:** accelerated aging, spruce wood, FTIR analysis, color, surface morphology, roughness and waviness

## Abstract

Spruce wood is widely used in outdoor applications, but its susceptibility to degradation under exposure to sunlight and moisture is a major concern. This study investigates the impact of accelerated aging on spruce wood’s surface chemistry, microstructure, geometry, and discoloration. The study was performed in two outdoor aging modes: dry and wet. The accelerated aging effects were evident in the changes in spruce wood structure, as well as in the other studied properties. During aging, it developed significant discoloration. Under simulated rainless outdoor conditions (dry mode), spruce wood gradually became dark brown. Under conditions involving rain (wet mode), the discoloration was qualitatively different from the dry mode. FTIR spectroscopy showed that during the accelerated aging of wood, lignin was mainly degraded, especially in the early stages of the process. A linear correlation was found between the changes in lignin and the color changes in the wood. There was an increase in carbonyl groups in the dry mode, which contributed to the color change and was also influenced by changes in extractives. The wet mode caused the leaching out of carbonyl groups. The observed decrease in cellulose crystallinity, together with the degradation of hydrophobic lignin, may result in the increased hydrophilicity of photodegraded wood. For both modes, there were different changes in the wood micro- and macrostructure, reflected in the surface morphology. The roughness increased during the aging process in both modes. The slightest changes in the roughness parameters were identified in the grain direction in the dry mode; the most evident was that the roughness parameters increased perpendicular to the grain in the wet mode. The demonstrated mechanism backing up the aging-related changes to the spruce wood structure and the relations unveiled between these changes and the changes in the spruce wood surface properties can provide an issue point for seeking ways how to mitigate the negative effects of the environmental factors the wood is exposed to.

## 1. Introduction

In outdoor environments, the surfaces of almost all materials are affected by various radiation types, moisture, temperature, and emissions, which act in interactions. This induces the gradual degradation of the exposed surfaces. Wood is not an exception. Wood, a natural composite material, exhibits unique aging behavior compared with other materials [1,2,3,4]. Wood has a characteristically complex surface morphology, backed up by its heterogeneous anatomical structure at the macro-, micro-, submicro-, and nano-levels [5,6]. In addition, wood material is hygrophilous, porous, and anisotropic, which is important for its degradation during the aging process. Diversely shaped and assembled anatomical elements of wood represent a heterogeneous porous system. Besides this, species-dependent differences in pore shape, size, mutual linking, and arrangement are obvious [5,7]. The porous nature of wood results in a large interior area and high surface roughness—mostly negative in the context of aging.

Wood degradation caused by the effects of various environmental factors is a very intricate and effort-demanding issue that needs a comprehensive approach. For a long time, the research on wood resistance against aging has been oriented on the variability of wood surface structure and properties, on degradation causal agents, on possibilities of how to avoid the degradation, and on the study of a range of factors supposed to influence the aging of wood surfaces [7,8,9,10,11,12,13,14,15]. The results of several works dealing with this problem have been summarized in [1,2].

Abiotic agents affecting a wood surface layer induce changes to its chemical structure and the associated changes in its properties [16,17]. Mi et al. [18] investigated the effects of natural weathering on aged wood from historic wooden buildings. They reported that all aged samples showed varying degrees of deterioration and that some aged samples showed a decrease in lignin content while cellulose content remained constant or increased. The decrease in lignin and hemicelluloses and the increase in cellulose content near the surface have been observed by several authors ([2] and references cited therein). Lignin strongly absorbs UV light and is photodegraded during both natural and accelerated aging. Lignin degradation during accelerated aging proceeds with the formation of *o*- and *p*-quinones, carbonyl groups and certain types of C=C bonds, such as stilbene derivatives. By comparing both the dry and wet processes, it was found that aging in the presence of water significantly enhanced the degradation of lignin. The degree of lignin degradation by dry exposure is depth-dependent. However, in the case of wet exposure, lignin degradation in the outermost cell walls proceeds from both the exposed surface side and the lumen side of the cell walls [19,20]. Lignin is most susceptible to UV light, but polysaccharides, especially hemicelluloses, can also be degraded via hydrolysis reactions to reduce the degree of polymerization, leading to chain shortening and even to the formation of oligosaccharides and monosaccharides [21]. Photodegradation forms new lignin chromophores, which cause discoloration and consist mainly of quinone-type structures [19].

The first degradation symptoms manifest in visual properties, and the first is discoloration due to photo-oxidation of polymers contained in wood, mainly lignin [9,10,13,22]. This is important evidence, especially from the viewpoint of targeted wood surface modification and treatment with transparent coating materials. The color change can also effectively indicate specific changes in wood chemistry [16]. The color spaces of the individual wood species have been recognized as virtually separated [23,24], and the supposition that the separations will be preserved after aging seems well-reasoned. It follows that the wood color variance during the aging process needs to be studied separately for each wood species.

During aging, the wood structure degradation is aggravated progressively, proceeding from the external wood surface down to a depth of several millimeters [11,25]. First, the middle lamella, consisting mostly of lignin, is degraded, and the delamination of wood cell elements takes place. The result is a plastic wood surface structure. This is mainly typical for coniferous wood species displaying differences in density between their early wood and late wood. Changes in wood structure are accompanied by changes in other surface properties [11,15,26,27,28].

Wood surface degradation during natural aging is caused by several factors (radiation, water, heat, wind, and pollutants) acting in interactions. Important is also the environment in which the wood is exposed to these factors. Thus, in the case of natural aging, it is not possible to examine the effects of the individual factors separately. Contrarily, accelerated aging simulated in a xenotest provides such a possibility. Under precisely fixed conditions, the influence of the radiation alone or the coupled influence of radiation and rain can be studied. This has been confirmed by Froidevaux and Navi [29].

This work aimed to disclose the chemical and physical background of aging-related degradation effects in native spruce wood. This wood is the most common material used in making constructions intended for outdoor conditions, and, as such, it is exposed to the negative effects of environmental agents. This study observed and evaluated the influence of radiation on the accelerated aging process and the coupled influence of radiation in the interaction with water on changes in spruce wood surface’s chemical composition, structure, geometry, and discoloration.

## 2. Materials and Methods

### 2.1. Experimental Material

**Figure 1:**
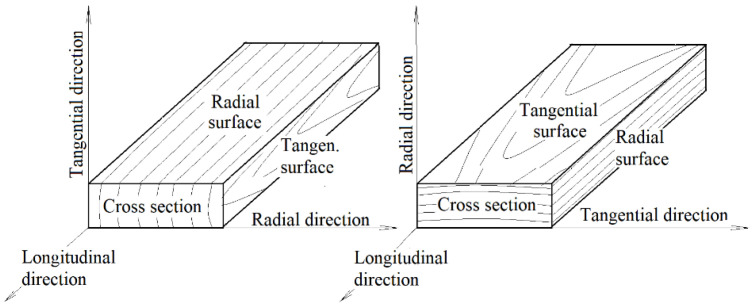
Prepared test specimens of spruce wood.

The degradation phenomena generated during the accelerated aging process were studied on radial and tangential surfaces of spruce wood specimens, each 100 mm × 50 mm × 15 mm (length × width × thickness) in size—see Figure 1. All the specimens were exposed to a relative air humidity of 65% and a temperature of 20 °C for one month to attain a wood moisture content of 12%. Before placing the specimens into the xenotest chamber, their relevant surface properties and body parameters (roughness and waviness profiles, chemical properties, and color) were measured. These values served as comparative standards for evaluating the changes in the studied properties measured during aging. Changes in wood surface structure and properties associated with accelerated aging were observed at intervals after 100, 200, 400, and 600 aging hours. For each aging mode, 15 radial and 15 tangential specimens were used.

### 2.2. Accelerated Aging

The accelerated wood aging was simulated in a xenotest chamber Q-SUN Xe-3-HS (Q-Lab Europe, Ltd., Bolton, UK). The experimental material was arranged equidistantly across the xenotest chamber. To ensure equal radiation intensity and heat for all the specimens, the specimens were regularly shifted according to the recommended schedule.

The aging conditions in the xenotest chamber followed the standard ASTM G 155 [30]. This standard is a fundamental one in determining the conditions for accelerated aging for non-metallic materials with the aid of a xenon discharge tube. Two modes for outdoor conditions were chosen: “dry mode” and “wet mode”. The first mode simulated outdoor conditions in the case of when wood is exposed to UV radiation but protected from rainwater; the second simulated the conditions when wood is exposed to both factors, UV and rain.

The radiation intensity was 0.35 W·m^–2^, with a radiation wavelength of 340 nm, following the standard. This value corresponds to the mean annual value for the temperate zone. The temperature, controlled on a black panel, corresponded to the maximum temperature on the panel surface. In both modes, one accelerated aging cycle consisted of two steps, covering 120 min altogether (Table 1 and Table 2). For each mode, the total duration of the aging process was 600 h, which was equivalent to 300 cycles.

### 2.3. Fourier Transform Infrared (FTIR) Spectroscopy

FTIR spectra of the wood surface were recorded on a Nicolet iS10 FT-IR spectrometer (Thermo Fisher Scientific, Waltham, MA, USA) equipped with Smart iTR using an Attenuated Total Reflectance (ATR) sampling accessory with a diamond crystal (Thermo Fisher Scientific). The spectra were recorded in an absorbance mode (A) from 4000 to 650 cm^−1^ with a spectral resolution of 4 cm^−1^, and 32 scans were used. Measurements were made on four replicates per sample. The peak at 1030 cm^−1^ (assigned to CO stretching) was used to normalize the obtained infrared spectra. This band shows little changes during wood photodegradation in a xenotest chamber [17]. Calculations for changes due to aging were made using the ratio of absorbances A_0_/A_t_, where A_0_ is the absorbance of the corresponding peak of the reference sample, and A_t_ is the absorbance of the peak at time t.

### 2.4. Color Measurement

The colorimetric values of the coordinates L*, a*, and b* on the referential and engraved specimens were measured with a spectrophotometer Spectro-guide 45/0 gloss (BYK-GARDNER GmbH, Geretsried, Germany). The measurements were taken at ten spots per specimen. The color differences ∆L*, ∆a*, and ∆b* under different irradiation conditions and the total color difference ∆*E* were determined according to the following equations:

(1) $${∆L}^{*}=L_{2}-L_{1},L_{3}-L_{1}\ldots ,L_{n}-L_{1}$$

(2) $${∆a}^{*}=a_{2}-a_{1},a_{3}-a_{1},\ldots ,a_{n}-a_{1}$$

(3) $${∆b}^{*}=b_{2}-b_{1},b_{3}-b_{1},\ldots ,b_{n}-b_{1}$$

(4) $$∆E=\sqrt{{∆L}^{2}+{∆a}^{2}+{∆b}^{2}}$$

Note: the “2 − *n*” means the color value after wood surface irradiation, and the index “1” denotes the referential value measured on the wood surface before the aging process.

For each mode, the color was measured at five spots on each specimen, so the total number of measurements for each surface treatment mode was 75.

### 2.5. Evaluation of Wood Surface Morphology

The surface morphology of the wood irradiated at the xenotest was evaluated at specified time intervals based on the roughness and waviness parameters: *R_a_* (mean arithmetic deviation) and *R_z_* (the maximum peak height plus the maximum depression depth within the cut-off, or a sampling length) and *Wa* (mean arithmetic deviation) and *Wt* (the maximum peak height plus the maximum depression depth within the entire evaluation length). The roughness and waviness profiles were recorded with the aid of a profilometer, Surfcom 130A (Carl Zeiss, Oberkochen, Germany), consisting of a measuring unit and an evaluation unit.

Roughness was measured on radial and tangential surfaces, parallel to the grain course and perpendicular to the grain course, at five different spots. The entire traversing length consisted of a pre-length segment, five sample length segments *l*r (cut-off λc), and a post-length segment *l*p. The basic lengths were chosen from the interval 0.025−8 mm based on the preliminary measured values of the roughness parameters *R_a_* and *R_z_*.

The degradation of the wood surface structure after having finished the aging process was studied with the aid of a SEM microscope.

### 2.6. Evaluation of the Results

The results were processed in the program STATISTICA version 10.0 (Dell Statistica, Prague, Czech Republic). The basic statistical characteristics (arithmetical mean, standard deviation) were determined using descriptive statistics. The impacts of the studied factors on the given properties were evaluated with the multi-way variance analysis (MANOVA) and Duncan’s test.

## 3. Results and Discussion

### 3.1. Wood Discoloration Induced by Accelerated Aging Process

The accelerated wood aging process induced considerable surface discoloration in spruce test specimens. This was true for both the dry and the wet modes. The results of the three-way variation analysis confirmed significant influences for all three factors investigated (aging mode, aging time, and irradiated surface type) and their interactions on spruce wood discoloration. The basic statistical characteristics for the individual color coordination values are shown in Table 3.

Before aging, the average values of lightness L* in the individual spruce sample sets ranged from 84 to 88 (Table 3). These values were in good accordance with the values reported for spruce wood in [31,32].

During the accelerated aging process simulating outdoor conditions without rain (dry mode), lightness significantly decreased with extending irradiation time. During aging, the values of the color coordinates a* and b* also varied (Table 3). In all cases, the a* and b* values were positive, which means that the a* values fitted the red zone, and the b* values ranged within the yellow zone, equally in good accordance with the above-cited authors. With the progressing aging time, the coordinate a* was getting more and more saturated. The coordinate b* exhibited significantly increasing saturation as far as 200 aging hours; then, no significant variation could be observed.

**Figure 2:**
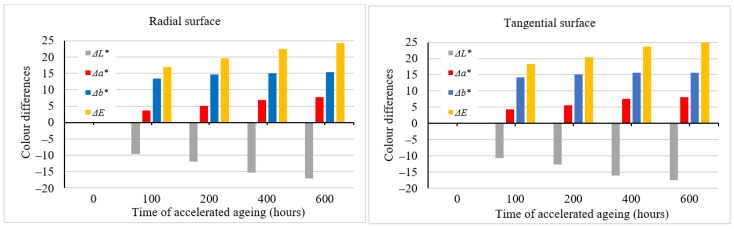
Differences in color coordinates and the total color difference during accelerated aging (dry mode).

The color variation generated by the effects of solar radiation simulated in the aging process could be expressed through the color coordinate differences ∆L*, ∆a*, and ∆b*; see Figure 2. The variations in color coordinates were also reflected in the total color difference ∆E*, with ∆E* > 12, which corresponds to the degree six of the six-degree discoloration scale [33], thus representing a different color hue compared with the original one. With further advances in aging time, the total color difference values increased, too, far beyond the value of 12.

**Figure 3:**
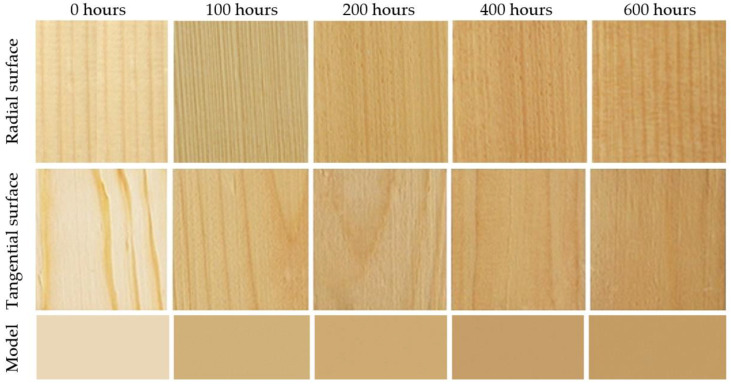
Color variation in spruce wood specimens during accelerated aging (dry mode).

The aging time-related color variations on the tangential and the radial surfaces were very similar, both in terms of quality and quantity. Despite the fact that statistically significant differences were confirmed between these two surface types, from a practical standpoint, they are unimportant. We suppose that these different color patterns are mainly due to the diverse heterogeneous structures across the radial and tangential surfaces. Spruce surface color variation during accelerated aging simulating outdoor conditions is also documented in Figure 3. Spruce wood belongs to light-colored wood species. In these species, the photodegradation causes more intensive darkening [9,10,13].

**Figure 4:**
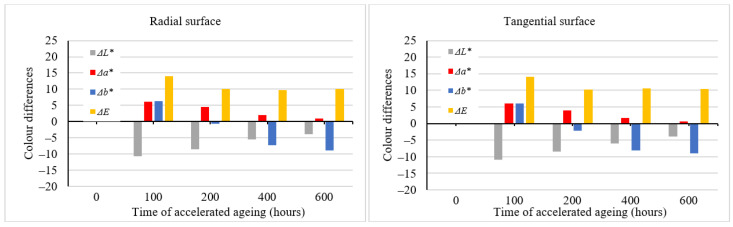
Differences in color coordinates and the total color difference during accelerated aging (wet mode).

**Figure 5:**
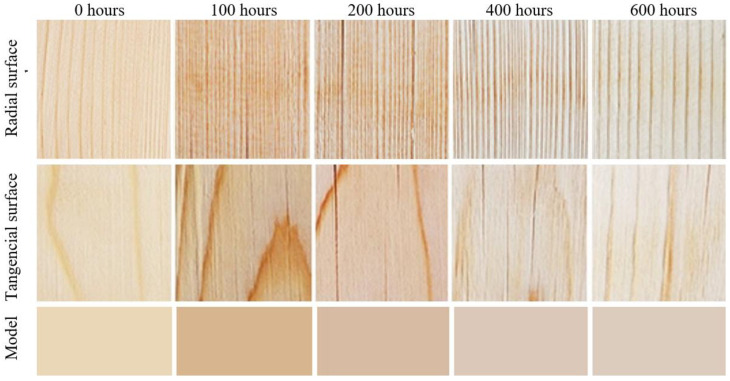
Color variation in spruce wood specimens during accelerated aging (wet mode).

During the accelerated aging process simulating outdoor conditions in the presence of rain (wet mode), the color variation was qualitatively different from the one associated with the dry mode. The lightness exhibited a conspicuous reduction after the first aging hours, with the coordinates a* and b* growing in saturation in the red and yellow areas, respectively (Table 3*)*. This resulted in coloring the spruce wood into darker brown. After 100 aging hours, the ∆E* values were >12 (Figure 4), corresponding again to the degree six of the six-degree scale [33]. After this moment, the interaction between radiation and water caused the wood surface color to fade step by step. After 600 aging hours, the lightness values were close to the original ones. The values of a* and b* decreased, too, shifting to achromatic colors. In this way, the wood obtained a brown-grey hue, the so-called patina. As the total color difference values ∆E* were close to 10, the final discoloration was found considerable thus far (degree five of a six-degree scale). Also, in this case, the aging time-dependent differences between the radial and the tangential surfaces were very similar in their quality and quantity and insignificant in terms of practical use. The color variation is also documented in Figure 5.

At relevant time intervals in the accelerated aging process, average values of color coordinates were measured experimentally on spruce wood surfaces. These values were then used as the input data for Adobe Photoshop to perform color simulation (Figure 3 and Figure 5—model). The figures illustrate good accordance between the factual specimens and the model obtained using the simulation.

### 3.2. FTIR Spectroscopy

**Figure 6:**
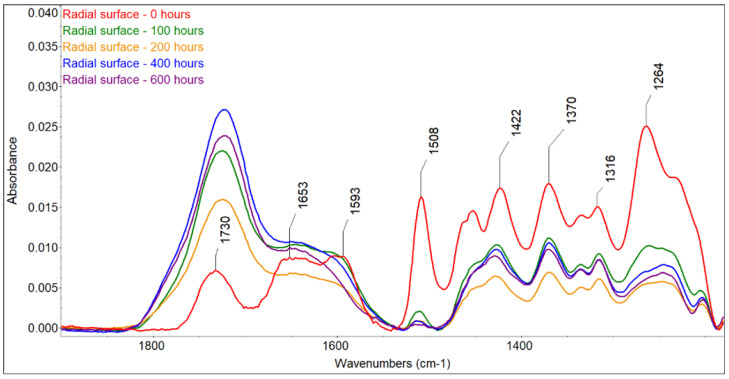
FTIR spectra of untreated and photodegraded spruce wood (radial surface, dry mode).

**Figure 7:**
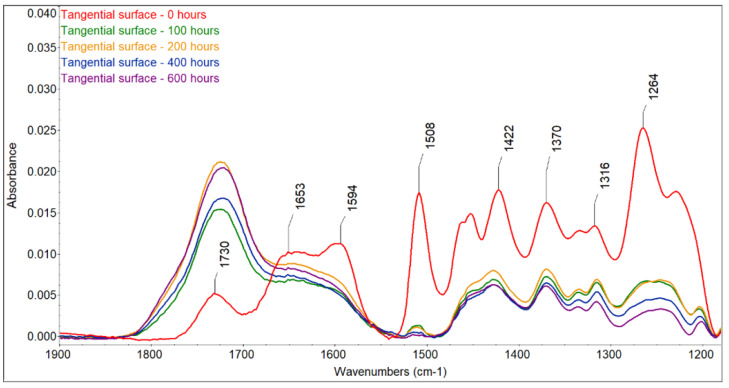
FTIR spectra of untreated and photodegraded spruce wood (tangential surface, dry mode).

**Figure 8:**
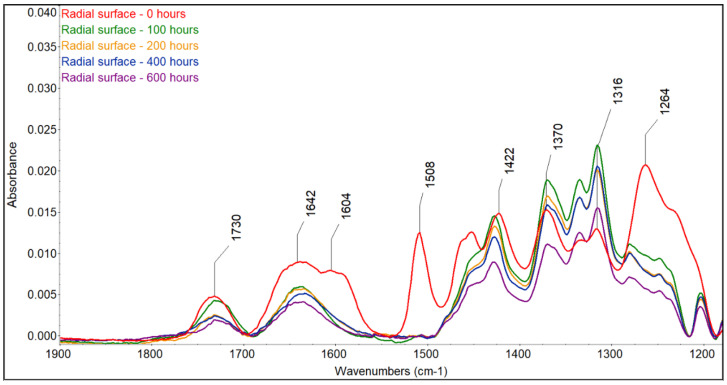
FTIR spectra of untreated and photodegraded spruce wood (radial surface, wet mode).

**Figure 9:**
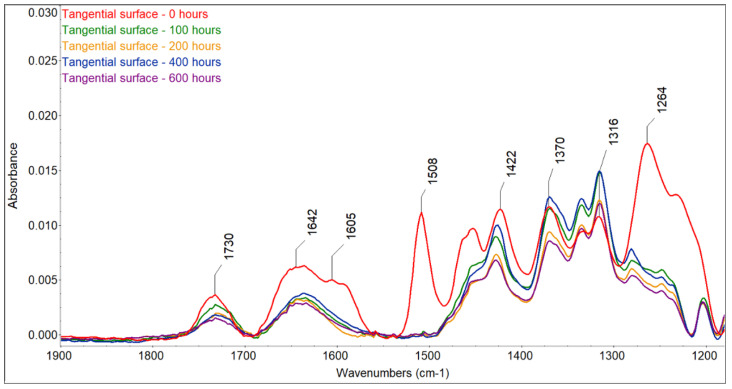
FTIR spectra of untreated and photodegraded spruce wood (tangential surface, wet mode).

The color changes observed for the two accelerated aging modes were caused by changes in the wood surface chemistry. FTIR spectra showed that UV radiation alone or in combination with water leaching causes significant changes on the surface of spruce wood (Figure 6, Figure 7, Figure 8 and Figure 9). The fingerprint region (1180–1900 cm^−1^) is shown in this study for clarity.

The most sensitive molecule is the lignin macromolecule because it is a good absorber of ultraviolet light. The photons of UV radiation can break the aromatic bonds of lignin. The produced free radicals react with oxygen to form carbonyl groups [34,35,36]. The characteristic peak for guaiacyl lignin (1508 cm^−1^) [37] decreased by approximately 98% of the original absorbance, with a greater decrease observed in the wet mode, where it completely disappeared. A sharp decrease occurred during the first 100 h of irradiation, and further accelerating aging had little effect on lignin changes. Similar results had been obtained by [38], who found a decrease in the peak at 1510 cm^−1^ to 60% of the original absorbance value when the spruce wood was irradiated with UV light. In addition to the decrease in the peak at 1508 cm^−1^, reduced absorbance was also observed around 1470 cm^−1^ (aromatic deformation of the C-H) and at 1264 cm^−1^ (guaiacyl ring breathing), which agrees with the results obtained with UV irradiation and the water leaching of Scots pine and spruce wood [39,40,41]. Several authors found a strong decrease in the absorbance of lignin in wood during the photodegradation process associated with the formation of carbonyl groups [38,41,42].

Changes in lignin were, therefore, accompanied by the formation of new carbonyl groups (1653 cm^−1^ − C=O conjugated and 1730 cm^−1^ − C=O unconjugated), which caused changes in the color of the wood. The formation of carbonyl groups indicates photoinduced oxidation of the wood surface. The cleavage of β-O-4 bonds in lignin leads to the formation of different types of chromophores, in particular quinones [38,43]. The growth of non-conjugated carbonyl groups was more pronounced in the early stages of irradiation. In contrast to the dry mode, where there was an increase in carbonyl groups (1653 cm^−1^ − C=O conjugated and 1730 cm^−1^ − C=O non-conjugated), in the wet mode of accelerated aging, we observed a decrease in both types of carbonyl groups due to the extraction of the formed carbonyl compounds with water, which is in line with the observation of [44,45].

**Figure 10:**
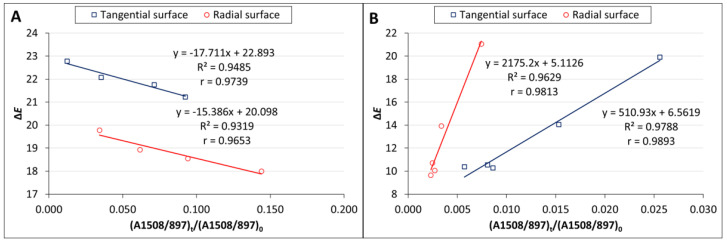
The dependence between lignin content and color changes. (**A**) dry mode, (**B**) wet mode.

Color change follows a similar pattern to lignin degradation, with the greatest changes occurring in the early stages of irradiation and being greater in the T direction than in the R direction. We found a linear correlation between lignin content and color changes with high correlation coefficients (from 0.9653 to 0.9893) (Figure 10) and a non-linear correlation between carbonyl groups at 1735 cm^−1^, which was also observed by Müller et al. [38]. These authors explain the linear dependence with the formation of quinones and the non-linear dependence with the formation of aliphatic carbonyl bonds. The photo-oxidation and oxidation of cellulose and hemicelluloses lead to the formation of the aldehyde and ketone groups on carbon atoms C2 and C3, which may contribute to the increased absorbance at wavelengths of 1653 and 1730 cm^−1^ [38], but the rate of change in cellulose is much lower than in lignin [13]. However, apart from lignin, the largest color changes during photodegradation can be caused by extractives. However, the content of extractives in wood is so small that, according to some authors [14,46], it is not possible to monitor their chemical changes using the FTIR method. On the other hand, Pandey [42], comparing unextracted and extracted UV-irradiated *Acacia auriculaeformis* wood, found that the photodegradation of polyphenolic extractives contributes to color changes. The different composition and content of extractives in individual woody plants can, therefore, influence color changes in the photodegradation process of the wood in different ways, which is consistent with several works [9,10,42]. Correlations between color changes and changes in the mechanical properties of wood have been found in several works. According to our research, this correlation applies mainly to the woody plants of the temperate zone, and even then, only if these woody plants do not have significant amounts and composition of extractives [47].

The ratio of absorbances A1370/A1508 can be used to calculate the wood crystallinity index (CI) [48]. The peak at 1370 cm^−1^ is characteristic of cellulose, and that at 1508 cm^−1^ is characteristic of lignin; in our experiments, a linear increase with high correlation coefficients (r = 0.92–0.98) occurred due to the faster degradation of amorphous lignin compared with mostly crystalline cellulose, and greater changes occurred in the wet mode compared with the dry mode (Table 4 and Table 5). Similar changes were also observed during the natural weathering of spruce wood [39] and the accelerated aging of pine wood [48].

FTIR spectroscopy can be used to study changes in cellulose, particularly changes in its crystallinity. The absorbance at 1420–1430 cm^−1^ is caused by skeletal vibrations associated with C-H planar deformations of cellulose and is attributed to its crystalline part. Bands at 1372–1375 cm^−1^ (CH deformation vibrations in cellulose and hemicelluloses) and 1160–1165 cm^−1^ (C-O-C asymmetric valence vibrations in cellulose and hemicelluloses) are characteristic of carbohydrates. The band at 898 cm^−1^ is caused by CH deformations in cellulose and its amorphous part [49,50,51,52].

The A1420/A898 ratio was proposed by Nelson and O’Connor [53] as an empirical crystallinity index (LOI—lateral order index), and the A1372/A2900 ratio as a total crystallinity index (TCI). Regarding the mobility of cellulose chains, hydrogen bond intensity (HBI) is closely related to the crystallinity of cellulose, as well as to the amount of bound water [49]. The band at 3400 cm^−1^ is due to −OH valence vibrations and at 1320 cm^−1^ to CH_2_ rocking vibrations in cellulose, and the A3400/A1320 ratio is used to study HBI in different wood species [51,54,55].

Accelerated aging causes a similar progression of LOI values, namely a decrease in the first stage of action, which is consistent with the results obtained in the photodegradation of deciduous and coniferous trees [56,57]. The decrease in TCI values accelerates in the later stages of photodegradation. The decrease in crystallinity increases the amount of amorphous cellulose, which leads to an increase in the hydrophilicity of aged wood. Changes in HBI values indicate the reorganization of hydrogen bonds or changes inbound water in cellulose.

### 3.3. Spruce Wood Surface Geometry and Morphology

The four-way variance analysis processing confirmed significant impacts for all four evaluated factors (aging mode, aging time, anatomical direction, and irradiated surface), as well as their interactions on the roughness parameters discussed.

The original spruce wood surface pre-treated with milling exhibited the lowest roughness values. As the unevenness of these surfaces overpassed 500 nm, they were considered surfaces with rough unevenness [58], and this is typical for wood. In most cases, significant differences in roughness between the radial and the tangential surfaces were not identified. Occasionally, significant differences in roughness parameters were confirmed; these, however, were not important from a practical viewpoint. Significantly lower roughness values that were measured in the grain direction than perpendicular to the grain were due to the orientation of the cell wall elements. The roughness data we obtained for the milled surface were in good accordance with the results reported in [59,60,61].

**Figure 11:**
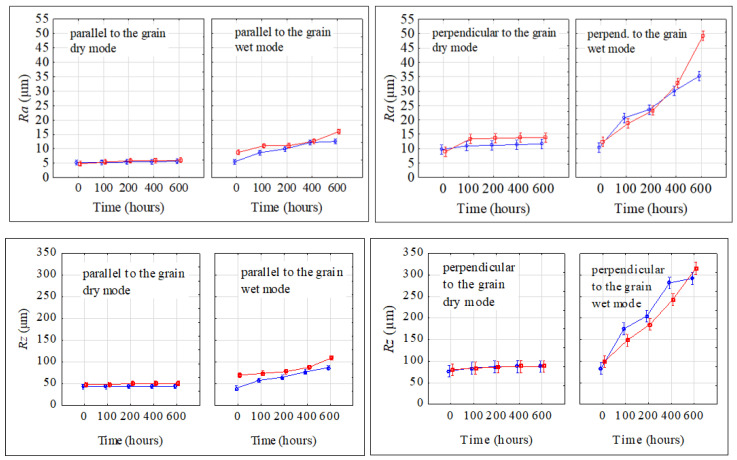
Parameters *R_a_* and *R_z_* varying during the accelerated aging process. 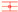 radial surface, 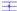 tangential surface.

The advancing aging process caused roughness to increase in both aging modes (Figure 11). This figure demonstrates that the smallest changes in roughness parameters were observed in the grain direction in the dry mode, and the highest increase in roughness parameters was found perpendicular to the grain in the wet mode. In this case, 600 aging hours resulted in more than a three-fold increase in the two roughness parameters. The aging-related changes in the roughness parameters *R_a_* and *R_z_* were practically identical on the radial and tangential surfaces.

**Figure 12:**
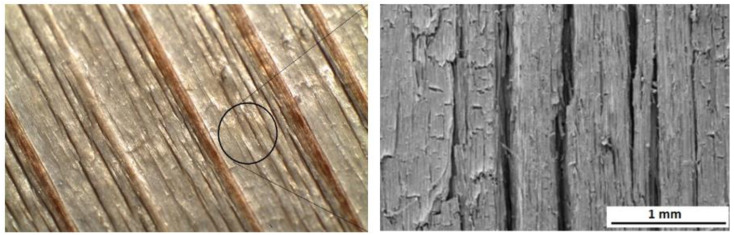
Morphology of radial spruce wood surface after 600 accelerated aging hours (wet mode).

The solar radiation and heat simulated in the dry aging mode caused depolymerization, primarily of lignin, and multiplication of carbonyl groups, but without their washing out. Consequently, the wood morphology remained without substantial changes. On the other hand, the wet mode, comprising radiation, oxidation, and heat in interactions with water, caused a decline in the carbonyl groups because of their extraction with water. Neither hydrolyzing reactions may be excluded. The hydrolysis mainly affects the acetyl groups in hemicelluloses [25]. All these phenomena resulted in more conspicuous wood surface degradation. Individual annual rings in spruce wood show characteristic differences in density between the early wood and the late wood, so the degradation primarily concerned early wood [62,63]. Moliński et al. [64] reported that the average density value for early spruce wood was about 300 kg·m^−3^, while for the late wood, it was 750 kg·m^−3^. This difference in density resulted in more extensive early wood erosion in the wood aging process and evident increases in roughness, measured mainly perpendicularly to the grain course. These differences in erosion extent between the early and late wood caused the forming of the so-called “plastic texture“ (Figure 12). This is typical for coniferous wood species [63,65].

In the dry mode, the wood moisture content was kept constant (4.5 ± 0.3%); accordingly, the dimensional changes were negligible. In the case of the wet mode, the average moisture content in the specimens subject to aging was significantly higher, ranging from 11% to 16%. Due to the steeper moisture gradient, the surface layers revealed restricted swelling or shrinkage. This probably enhanced the frequency occurrence of surface cracks observed in the case of the wet mode equally on the radial and the tangential surfaces (Figure 12).

**Figure 13:**
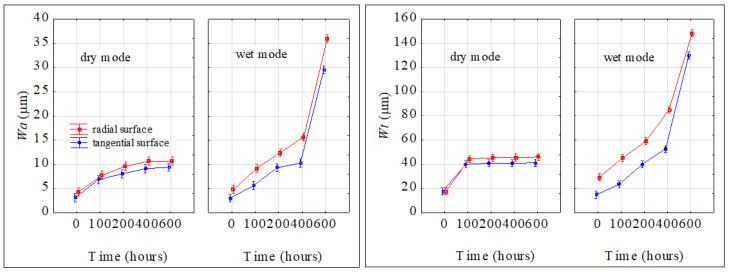
Parameters *Wa* and *Wt* varying during accelerated aging process (perpendicular to the grain).

Waviness was evaluated only perpendicularly to the grain. Also, for waviness, the results of the three-way variance analysis confirmed the important effects of all three studied factors (aging mode, aging time, and irradiated surface) and their interactions on the waviness parameters *Wa* and *Wt*. The waviness parameters varied with the aging time both in dry and wet modes (Figure 13). The two parameters significantly increased with prolonged aging time. Evidently, bigger changes were observed for the wet mode. While in the case of dry mode, the *Wa* values, after accomplishing the aging process, were found to be 2.5 to 3 times bigger than the original ones, in the case of wet mode, the corresponding increase was from 7.5- to 10-fold. Similar qualitative and quantitative changes during the aging process were also observed for the parameter *Wt*. Higher waviness was observed on radial surfaces.

**Figure 14:**
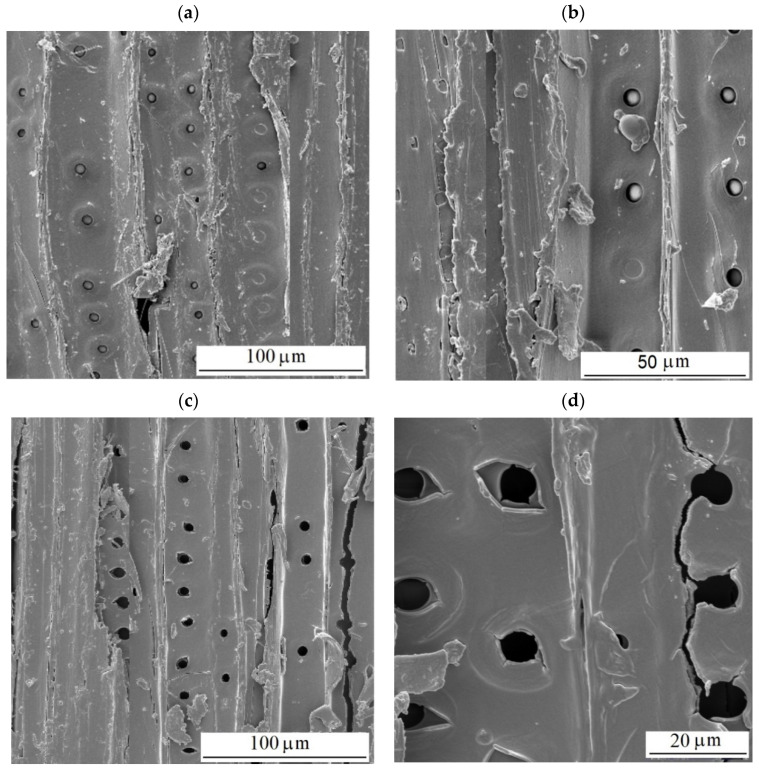

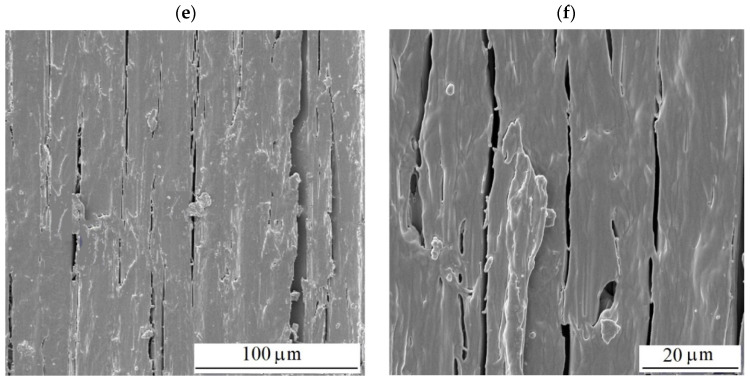
Structure of milled radial surfaces of spruce wood. (**a**,**b**) Before aging. (**c**–**f**) After 600 aging hours—dry mode.

The changes in the chemistry and morphology of the spruce wood surface during the aging process were also reflected through the changes in this microscopic wood structure. Before the aging, the cell elements in the milled spruce wood surfaces created a compact structure. The tracheids were cross-linked by means of undamaged middle lamellae. The inner surfaces of cross-cut tracheids exhibited intact bordered pits pair (Figure 14a,b). In the dry mode, after 600 aging hours, surficial cracks were observed; the bonds between the cell elements, however, were mostly preserved sufficiently compact (Figure 14c–f). The inner surfaces of early tracheids exhibited cracks in cell walls and in neighborhoods of bordered pits. These were, to a certain extent, distorted and lacking toruses. The character of cell walls destruction indicates their fragility aggravated due to radiation effects. On the other hand, the surfaces of intact, non-cut late wood tracheids manifested the occurrence of melt mass, identified, based on our chemical analyses, as lignin and lignin derivatives (Figure 14e,f). A similar phenomenon was observed by Ko et al. [66] during beech wood pre-treatment with hot water.

**Figure 15:**
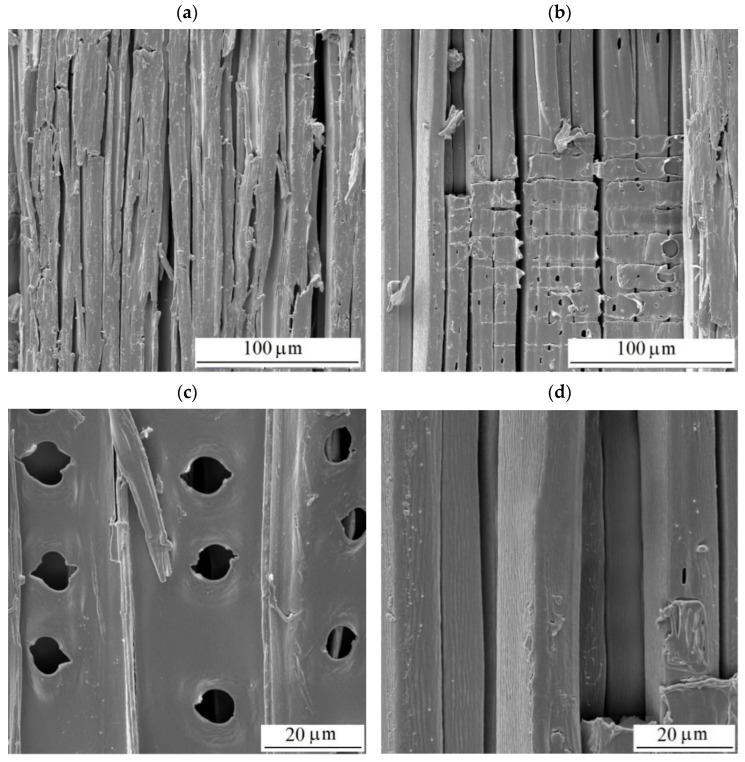
Structure of milled radial surfaces of spruce wood (**a**–**d**) after 600 aging hours—wet mode.

More conspicuous changes in the structure were observed after having accomplished the aging in the wet mode (Figure 15). After 600 aging hours, the surfaces exhibited more abundant cracks (Figure 15a,b). From many spots, the compound middle lamellae (Figure 15d), consisting mainly of lignin, were eroded. The removal of the middle lamellae considerably debilitated the connections between the tracheids. The supplied liquid water detached the tracheids from the surface step by step, and the surface roughness was progressively pronounced. The bordered pits were degraded, too. The pores were extended, and the toruses were eliminated totally or partially as the result of margin degradation (Figure 15c). The surfaces did not exhibit melted lignin because, unlike in the dry mode, this substance was also washed away with water. A similar form of surface degradation has been documented for several tropical woods subject to natural and accelerated aging [67].

## 4. Conclusions

The analysis of the experimental results has allowed us to draw the following conclusions.

- Sunlight exposure was a major factor in spruce wood discoloration. As early as after 100 irradiation hours, the total color difference values were ∆E* > 12, thus representing a new wood color hue, different from the original one.
- The interaction of sunlight with water during aging resulted in qualitatively different discoloration compared with the dry mode, leading to the formation of a patina on the wood surface.
- Water leaching during UV photodegradation increased the hydrophilicity of spruce wood by leaching out carbonyl groups. The accelerated aging of wood causes faster degradation of lignin compared with cellulose, and greater changes occurred in the wet mode compared with the dry mode.
- Spruce tracheids showed increased fragility with aging. Wet mode aging led to irreversible hydrogen bonding (hornification), impacting microstructural damage differently compared with dry mode aging.
- The compound middle lamella was eroded at many spots, which resulted in a significant decline in the coherence of the tracheids.
- The direction of wood fibers influenced the extent to which the surface roughness was affected by aging, with moisture exposure (wet mode) potentially amplifying this effect.

## Figures and Tables

**Table 1:** The aging parameters set according to the Standard ASTM G 155 “dry mode”.

Step	Mode	Radiation Intensity (W/m^2^)	Black Panel Temperature (°C)	Air Temperature (°C)	Relative Air Humidity (%)	Time (min.)
1	Radiation	0.35	63	48	30	102
2	Radiation-free	-	-	38	–	18

**Table 2:** The aging parameters set according to the Standard ASTM G 155 “wet mode”.

Step	Mode	Radiation Intensity (W/m^2^)	Black Panel Temperature (°C)	Air Temperature (°C)	Relative Air Humidity (%)	Time (min.)
1	Radiation	0.35	63	48	30	102
2	Radiation + water spraying	0.35	63	48	90	18

**Table 3:** Basic statistical characteristics of color coordinate values L*, a*, and b* for spruce wood at different phases of accelerated aging (number of measurements for each sample set n = 75).

Surface	Color Coordinates	Basic Statistical Characteristics	Aging Time (Hours)				
			0	100	200	400	600
			Dry Mode				
Radial	L*	$\bar{x}$	83.82	74.10	71.88	68.57	66.71
		*s*	1.67	1.26	1.22	1.20	1.18
	a*	$\bar{x}$	3.82	7.46	8.92	10.74	11.55
		*s*	0.70	0.35	0.36	0.34	0.31
	b*	$\bar{x}$	20.57	33.94	35.29	35.66	36.00
		*s*	0.71	0.60	0.68	0.87	0.89
Tangential	L*	$\bar{x}$	84.46	73.70	71.80	68.42	66.82
		*s*	1.65	1.57	1.24	1.36	1.40
	a*	$\bar{x}$	3.34	7.73	8.96	10.91	11.46
		*s*	0.72	0.62	0.57	0.58	0.60
	b*	$\bar{x}$	20.01	34.11	35.14	35.71	35.60
		*s*	1.76	0.90	0.69	1.07	0.95
			Wet mode				
Radial	L*	$\bar{x}$	87.19	76.44	78.52	81.72	83.30
		*s*	0.61	1.28	1.40	1.52	1.63
	a*	$\bar{x}$	2.84	8.97	7.34	4.79	3.67
		*s*	0.29	0.57	0.70	0.55	0.53
	b*	$\bar{x}$	18.04	24.31	17.32	10.77	9.10
		*s*	0.69	1.69	1.96	2.04	1.95
Tangential	L*	$\bar{x}$	87.71	76.81	79.19	81.63	83.76
		*s*	1.16	2.01	2.46	3.19	3.06
	a*	$\bar{x}$	2.42	8.63	6.35	4.09	3.09
		*s*	0.45	0.86	1.04	0.81	0.66
	b*	$\bar{x}$	17.56	23.58	15.32	9.54	8.63
		*s*	2.05	2.59	2.80	2.01	1.85

**Table 4:** The values of TCI, LOI, HBI, and CI (dry mode).

Time (h)	Radial Surface				Tangential Surface			
	TCI	LOI	HBI	CI	TCI	LOI	HBI	CI
0	2.52	1.02	1.63	1.10	2.40	1.32	1.85	0.93
100	2.49	0.72	1.99	5.55	2.25	0.68	1.66	6.00
200	2.46	0.74	1.77	8.73	2.19	0.73	1.85	8.10
400	2.39	0.74	2.30	13.46	2.14	0.70	1.92	15.61
600	2.11	0.79	1.92	26.22	1.94	0.83	2.44	50.00

**Table 5:** The values of TCI, LOI, HBI, and CI (wet mode).

Time (h)	Radial Surface				Tangential Surface			
	TCI	LOI	HBI	CI	TCI	LOI	HBI	CI
0	2.24	1.24	1.95	1.22	2.64	1.73	1.79	1.05
100	2.00	0.86	1.78	313.33	2.02	0.88	1.46	44.23
200	2.11	0.85	1.75	384.09	2.02	1.06	1.46	93.00
400	2.11	0.90	1.69	493.75	2.08	1.12	1.60	120.88
600	2.18	1.16	1.54	555.00	2.13	1.31	1.27	170.00

## Data Availability

Data are contained within the article.

## References

[1] Cogulet A., Blanchet P., Landry V. (2017). The Multifactorial Aspect of Wood Weathering: A Review Based on a Holistic Approach of Wood Degradation Protected by Clear Coating. BioResources.

[2] Kropat M., Hubbe M.A., Laleicke F. (2020). Natural, Accelerated, and Simulated Weathering of Wood: A Review. BioResources.

[3] Yona A.M.C., Žigon J., Matjaž P., Petrič M. (2021). Potentials of Silicate-Based Formulations for Wood Protection and Improvement of Mechanical Properties: A Review. Wood Sci. Technol..

[4] Gaff M., Kačík F., Gašparík M. (2019). Impact of Thermal Modification on the Chemical Changes and Impact Bending Strength of European Oak and Norway Spruce Wood. Compos. Struct..

[5] Sandberg D., Kutnar A., Karlsson O., Jones D. (2021). Wood Modification Technologies: Principles, Sustainability, and the Need for Innovation.

[6] Wagenführ R. (2007). Holzatlas.

[7] Golovin Y.I., Gusev A.A., Golovin D.Y., Matveev S.M., Vasyukova I.A. (2022). Multiscale Mechanical Performance of Wood: From Nano- to Macro-Scale across Structure Hierarchy and Size Effects. Nanomaterials.

[8] Rowell R.M. (2021). Understanding Wood Surface Chemistry and Approaches to Modification: A Review. Polymers.

[9] Liu X., Timar M.C., Varodi A.M., Nedelcu R., Torcătoru M.-J. (2022). Colour and Surface Chemistry Changes of Wood Surfaces Coated with Two Types of Waxes after Seven Years Exposure to Natural Light in Indoor Conditions. Coatings.

[10] Vidholdová Z., Reinprecht L., Pánek M. (2023). The Effect of Outdoor Weathering of Thermally Modified Spruce and Pine Woods on Their Surface Properties. Acta Fac. Xylologiae Zvolen.

[11] Huang X., Kocaefe D., Kocaefe Y., Boluk Y., Pichette A. (2012). A Spectrocolorimetric and Chemical Study on Color Modification of Heat-Treated Wood during Artificial Weathering. Appl. Surf. Sci..

[12] Gurău L., Timar M.C., Coșereanu C., Cosnita M., Stanciu M.D. (2023). Aging of Wood for Musical Instruments: Analysis of Changes in Color, Surface Morphology, Chemical, and Physical-Acoustical Properties during UV and Thermal Exposure. Polymers.

[13] Wang Y., Huang Y., Xue J., Peng Y., Cao J. (2022). Effects of heat treatment temperatures on the photostability of Moso bamboo during accelerated UV weathering. Wood Mater. Sci. Eng..

[14] Preklet E., Tolvaj L., Tsuchikawa S., Varga D. (2021). Photodegradation Properties of Earlywood and Latewood Spruce Timber Surfaces. Acta Silv. Lignaria Hung.

[15] Rubino G., Lo Monaco A., Lanteri L., Pelosi C. (2023). Improving the Technical Characteristics of Untreated and Heat-Treated Ayous Wood against Accelerating Ageing by Testing Two Application Modalities of an Innovative Polyurethane Coating for Outdoor Uses. Coatings.

[16] Peters F.B., Rapp A.O. (2021). Wavelength-dependent photodegradation of wood and its effects on fluorescence. Holzforschung.

[17] Colom X., Carrillo F., Nogués F., Garriga P. (2003). Structural Analysis of Photodegraded Wood by Means of FTIR Spectroscopy. Polym. Degrad. Stab..

[18] Mi X., Li Y., Qin X., Li J. (2023). Effects of Natural Weathering on Aged Wood from Historic Wooden Building: Diagnosis of the Oxidative Degradation. Herit. Sci..

[19] Kanbayashi T., Kataoka Y., Ishikawa A., Matsunaga M., Kobayashi M., Kiguchi M. (2018). Confocal Raman Microscopy Reveals Changes in Chemical Composition of Wood Surfaces Exposed to Artificial Weathering. J. Photochem. Photobiol. B.

[20] Kránitz K., Sonderegger W., Bues C.-T., Niemz P. (2016). Effects of Aging on Wood: A Literature Review. Wood Sci. Technol..

[21] Ghavidel A., Scheglov A., Karius V., Mai C., Tarmian A., Vioel W., Vasilache V., Sandu I. (2020). In-Depth Studies on the Modifying Effects of Natural Ageing on the Chemical Structure of European Spruce (*Picea abies*) and Silver Fir (*Abies alba*) Woods. J. Wood Sci..

[22] Kúdela J., Javorek L., Mrenica L. (2016). Influence of Milling and Sanding on Beech Wood Surface Properties. Part I. Surface Morfology. Ann. Wars. Univ. Life Sci.-SGGW Land Reclam..

[23] Hrčka R. (2008). Identification of Discoloration of Beech Wood In CIELAB Space. Wood Res..

[24] Olsson S.K., Johansson M., Westin M., Östmark E. (2014). Reactive UV-Absorber and Epoxy Functionalized Soybean Oil for Enhanced UV-Protection of Clear Coated Wood. Polym. Degrad. Stab..

[25] Reinprecht L. (2016). Wood Deterioration, Protection and Maintenance.

[26] Kúdela J., Mrenica L., Čunderlík I., Šmíra P. (2015). Morphological Changes on Spruce Wood Surface, Induced by Treatment with Dry Ice. Mater. Sci. Forum.

[27] Song J., Zhao J., Deng J., Lu S., Hang G., Zhang Y., Shu C.-M. (2023). Effect Mechanism of Dry and Wet Alternate Ageing on Wood during Exothermic Behaviour. J. Therm. Anal. Calorim..

[28] Tolvaj L., Persze L., Albert L. (2011). Thermal Degradation of Wood during Photodegradation. J. Photochem. Photobiol. B.

[29] Froidevaux J., Navi P. (2013). Aging Law of Spruce Wood. Wood Mater. Sci. Eng..

[30] (2005). Practice for Operating Xenon Arc Light Apparatus for Exposure of Non-Metallic Materials.

[31] Sikora A., Kačík F., Gaff M., Vondrová V., Bubeníková T., Kubovský I. (2018). Impact of Thermal Modification on Color and Chemical Changes of Spruce and Oak Wood. J. Wood Sci..

[32] Kúdela J., Andrejko M., Kubovský I. (2023). The Effect of CO_2_ Laser Engraving on the Surface Structure and Properties of Spruce Wood. Coatings.

[33] Allegretti O., Travan L., Cividini R. Drying Techniques to Obtain White Beech. Proceedings of the Wood EDG Conference.

[34] Ouadou Y., Aliouche D., Thevenon M.-F., Djillali M. (2017). Characterization and Photodegradation Mechanism of Three Algerian Wood Species. J. Wood Sci..

[35] Sandak A., Sandak J., Noël M., Dimitriou A. (2021). A Method for Accelerated Natural Weathering of Wood Subsurface and Its Multilevel Characterization. Coatings.

[36] Zborowska M., Stachowiak-Wencek A., Nowaczyk-Organista M., Waliszewska B., Prądzyński W. (2015). Analysis of Photodegradation Process of Pinus Sylvestris L. Wood after Treatment with Acid and Alkaline Buffers and Light Irradiation. BioResources.

[37] Hofmann T., Tolvaj L., Visi-Rajczi E., Varga D. (2022). Chemical Changes of Steamed Timber during Short-Term Photodegradation Monitored by FTIR Spectroscopy. Eur. J. Wood Wood Prod..

[38] Müller U., Rätzsch M., Schwanninger M., Steiner M., Zöbl H. (2003). Yellowing and IR-Changes of Spruce Wood as Result of UV-Irradiation. J. Photochem. Photobiol. B.

[39] Mastouri A., Azadfallah M., Kamboj G., Rezaei F., Tarmian A., Efhamisisi D., Mahmoudkia M., Corcione C.E. (2023). Kinetic Studies on Photo-Degradation of Thermally-Treated Spruce Wood during Natural Weathering: Surface Performance, Lignin and Cellulose Crystallinity. Constr. Build. Mater..

[40] Bhagia S., Ďurkovič J., Lagaňa R., Kardošová M., Kačík F., Cernescu A., Schäfer P., Yoo C.G., Ragauskas A.J. (2022). Nanoscale FTIR and Mechanical Mapping of Plant Cell Walls for Understanding Biomass Deconstruction. ACS Sustain. Chem. Eng..

[41] Preklet E., Tolvaj L., Visi-Rajczi E., Hofmann T. (2021). Effect of Water Leaching on Photodegraded Scots Pine and Spruce Timbers Monitored by FTIR Spectroscopy. Forests.

[42] Pandey K.K. (2005). A Note on the Influence of Extractives on the Photo-Discoloration and Photo-Degradation of Wood. Polym. Degrad. Stab..

[43] Bergamasco S., Zikeli F., Vinciguerra V., Sobolev A.P., Scarnati L., Tofani G., Scarascia Mugnozza G., Romagnoli M. (2023). Extraction and Characterization of Acidolysis Lignin from Turkey Oak (*Quercus cerris* L.) and Eucalypt (*Eucalyptus camaldulensis* Dehnh.) Wood from Population Stands in Italy. Polymers.

[44] Bejo L., Tolvaj L., Kannar A., Preklet E. (2019). Effect of Water Leaching on Photodegraded Spruce Wood Monitored by IR Spectroscopy. J. Photochem. Photobiol. Chem..

[45] Doczekalska B., Stachowiak-Wencek A., Roszyk E., Sydor M. (2024). Thermochemical modification of beech wood with ammonium hydroxide. Eur. J. Wood Prod..

[46] Tolvaj L., Molnar Z., Nemeth R. (2013). Photodegradation of Wood at Elevated Temperature: Infrared Spectroscopic Study. J. Photochem. Photobiol. B.

[47] Kačík F., Kubovský I., Bouček J., Hrčka R., Gaff M., Kačíková D. (2023). Colour and Chemical Changes of Black Locust Wood during Heat Treatment. Forests.

[48] Lionetto F., Del Sole R., Cannoletta D., Vasapollo G., Maffezzoli A. (2012). Monitoring Wood Degradation during Weathering by Cellulose Crystallinity. Materials.

[49] Oh S.Y., Yoo D.I., Shin Y., Kim H.C., Kim H.Y., Chung Y.S., Park W.H., Youk J.H. (2005). Crystalline Structure Analysis of Cellulose Treated with Sodium Hydroxide and Carbon Dioxide by Means of X-ray Diffraction and FTIR Spectroscopy. Carbohydr. Res..

[50] Oh S.Y., Yoo D.I., Shin Y., Seo G. (2005). FTIR Analysis of Cellulose Treated with Sodium Hydroxide and Carbon Dioxide. Carbohydr. Res..

[51] Poletto M., Zattera A.J., Santana R.M.C. (2012). Structural Differences between Wood Species: Evidence from Chemical Composition, FTIR Spectroscopy, and Thermogravimetric Analysis. J. Appl. Polym. Sci..

[52] Prasetia D., Purusatama B.D., Kim J.-H., Jang J.-H., Park S.-Y., Lee S.-H., Kim N.H. (2024). X-ray Diffraction, Fourier Transform Infrared Spectroscopy, and Thermal Decomposition Analyses of Virgin Cork Elements in Quercus Variabilis Grown in Korea. Wood Sci. Technol..

[53] Nelson M.L., O’Connor R.T. (1964). Relation of Certain Infrared Bands to Cellulose Crystallinity and Crystal Latticed Type. Part I. Spectra of Lattice Types I, II, III and of Amorphous Cellulose. J. Appl. Polym. Sci..

[54] Åkerholm M., Hinterstoisser B., Salmén L. (2004). Characterization of the Crystalline Structure of Cellulose Using Static and Dynamic FT-IR Spectroscopy. Carbohydr. Res..

[55] Schwanninger M., Rodrigues J.C., Pereira H., Hinterstoisser B. (2004). Effects of Short-Time Vibratory Ball Milling on the Shape of FT-IR Spectra of Wood and Cellulose. Vib. Spectrosc..

[56] Tribulová T., Kačík F., Evtuguin D.V., Čabalová I., Ďurkovič J. (2019). The Effects of Transition Metal Sulfates on Cellulose Crystallinity During Accelerated Ageing of Silver Fir Wood. Cellulose.

[57] Huang X., Kocaefe D., Kocaefe Y., Boluk Y., Krause C. (2013). Structural Analysis of Heat-Treated Birch (*Betule Papyrifera*) Surface during Artificial Weathering. Appl. Surf. Sci..

[58] Liptáková E., Kúdela J., Bastl Z., Spirovová I. (1995). Influence of Mechanical Surface Treatment of Wood on the Wetting Process. Holzforschung.

[59] Kúdela J., Mrenica L., Javorek L. (2018). The Influence of Milling and Sanding on Wood Surface Morphology. Acta Fac. Xylologiae.

[60] Gurau L. (2007). Quantitative Evaluation of the Sanding Quality in Furniture Manufacturing.

[61] Csanády E., Magoss E., Tolvaj L. (2015). Quality of Machined Wood Surfaces.

[62] Temiz A., Yildiz U.C., Aydin I., Eikenes M., Alfredsen G., Çolakoglu G. (2005). Surface Roughness and Color Characteristics of Wood Treated with Preservatives after Accelerated Weathering Test. Appl. Surf. Sci..

[63] Feist W.C. (1989). Outdoor Wood Weathering and Protection. Archaeological Wood.

[64] Moliński W., Cunderlik I., Krauss A., Fabisiak E., Jurek P. (2009). Gradient of Density and Tensile Strength along the Grains of Spruce Wood (*Picea abies*) within Individual Annual Rings. Ann. Wars. Univ. Life Sci.-SGGW For. Wood Technol..

[65] Rowell R.M. (2021). Handbook of Wood Chemistry and Wood Composites.

[66] Ko J.K., Kim Y., Ximenes E., Ladisch M.R. (2015). Effect of Liquid Hot Water Pretreatment Severity on Properties of Hardwood Lignin and Enzymatic Hydrolysis of Cellulose. Biotechnol. Bioeng..

[67] Mamoňová M., Reinprecht L. (2020). The Impact of Natural and Artificial Weathering on the Anatomy of Selected Tropical Hardwoods. IAWA J..

